# The Application of the Metacognitive Model of Desire Thinking and Craving in Problematic Social Networking Sites Use

**DOI:** 10.1007/s11126-023-10059-2

**Published:** 2023-10-21

**Authors:** Sara Bocci Benucci, Benedetta Tonini, Giulia Roffo, Silvia Casale, Giulia Fioravanti

**Affiliations:** 1 Department of Experimental and Clinical Medicine, University of Florence, Largo Brambilla, 3, 50134 Florence, Italy; 2 School of Psychology, University of Florence, Via della Torretta, 16, 50137 Florence, Italy; 3 Department of Health Sciences, Psychology Unit, Via San Salvi 12- Padiglione 26, 50135 Florence, Italy

**Keywords:** Craving, Desire thinking, FoMo, Metacognitions, Problematic Social Networking Sites Use

## Abstract

Cognitive models of addictive behaviours have highlighted the central role of Desire Thinking (DT) – a conscious and voluntary cognitive process orienting to prefigure images and information about a positive target-related experience – in increasing craving and maintaining addictive behaviors. The metacognitive model of DT and craving posits that metacognition plays a central role in understanding dysregulation in DT. The current study aims to test the role of metacognitions about DT, DT, and craving in the relationship between Fear of Missing Out (FoMo), boredom proneness, negative emotional reactivity and Problematic Social Network Sites Use (PSNSU). A sample of 529 participants (M_age_= 32.45 ± 13.33; F = 62.9%) completed an online survey. The hypothesised model produced an adequate fit to the data and accounted for 86% of PSNSU variance. FoMO predicted positive metacognitions about DT (PMDT), which predicted DT that, in association with craving, predicted PSNSU. Boredom proneness positively predicted PSNSU directly and indirectly through the serial mediation of PMDT, DT, and craving. A direct path between negative emotional reactivity and PSNSU was found. The current findings provide preliminary evidence for applying the metacognitive model of DT and craving in PSNSU. PMDT and DT may be central cognitive processes in craving and PSNSU for individuals who experience boredom proneness and FoMo.

## Introduction

Social network sites (SNSs) can be defined as websites that, relying on Internet-based technologies, offer various kinds of services to users connecting to the network itself, the most important of which is the management of users’ interpersonal relations on the Web. Most social networks have common features that can be identified in three main elements: the creation of a personal profile (public or semi-public) containing self-descriptive information, the creation of a list of friends, representing online social contacts of varying importance and strength, the exploration of one’s network and that of one’s friends, through a stream (e.g., Facebook’s News Feed) that is continuously updated with respect to the contents published by one’s contacts [1, 2]. The use of SNSs has boomed during the last two decades. In 2022, over 5 billion people were using SNSs worldwide, and the average daily SNSs usage was 147 min daily. Leading social media usage reasons worldwide are keeping in touch with friends and family, filling spare time, reading news stories, and posting about their life.

Despite the various benefits of SNSs use, including the possibility of establishing new offline relationships (initiated online), strengthening existing ones (an increase of social capital), providing social support when offline interactions are impossible or scarce, and collaborating with others, creating and organizing social groups or movements, several studies have highlighted potential negative consequences of excessive SNSs usage, like a decrease in involvement in real-life communities and worse academic performance, as well as relationship difficulties and mental health problems, like anxiety, depression, and body image disturbance (for reviews see [3–7]). In the excessive form, SNSs use is most notably associated with depression and anxiety [8].

Problematic SNSs use (PSNSU) has been defined as a lack of self-regulation in one’s use of SNSs that leads to negative consequences in everyday life [9]. Several scholars (e.g., [3]) argue that SNSs use may be addictive because some individuals experience symptoms like those experienced by persons who suffer from other forms of addiction. On the basis of the six components model proposed by Griffiths [10] PSNSU is defined as a combination of salience (the activity becomes the most important activity in the person’s life), tolerance (the need to spend an increased amount of time on SNSs to achieve the former effects), mood modification (the subjective experience that people report as a consequence of engaging in SNSs use), relapse (the tendency for repeated reversions to earlier patterns of SNSs use), withdrawal (feeling troubled when unable to use SNSs), and conflict (negative impacts of excessive usage of SNSs on the user’s life). Moreover, some authors have suggested that problematic use of social networks may fit with the ICD-11 category “other specified disorder due to addictive behaviors” [11]. This suggestion is based on empirical evidence highlighting that: (i) this behavioral pattern leads to clinically significant distress and impairments in everyday life; (ii) addiction framework theories can appropriately explain this behavior; and (iii) some of the mechanisms involved share similarities to those in other addictive behaviors (e.g., [12–14]). Although the scientific debate regarding the possibility of considering PSNSU as an addictive behavior is still open, some evidence that PSU shows similarities with substance and behavioral addictions emerged. Mood modification, salience, withdrawal symptoms, conflict, and relapse appear present in those who use SNSs excessively (e.g., [15]).

Regarding the prevalence of PSNSU, a meta-analysis conducted on 63 independent samples with 34,798 respondents from 32 nations spanning seven world regions revealed a pooled social media addiction prevalence of 24% [16]. Variations in prevalence among studies adopting distinct classification schemes were found: from 5% for studies adopting monothetic or strict monothetic classifications to 25% for studies adopting a cutoff for moderate-level or polythetic classifications. Moreover, cross-cultural comparisons revealed the pooled prevalence estimate obtained in collectivist nations to be twofold higher than that obtained in individualist nations (31% vs. 14% respectively). Another recent meta-analysis found that PSNSU is higher in low-income countries and the prevalence does not vary depending on age and gender [17].

The impact and the high prevalence of PSNSU encouraged an examination of the contributing factors to this problematic behavior in the last three decades [18, 19]. As evidenced by recent meta-analyses, personality factors related to PSNSU encompass neuroticism, conscientiousness [20, 21], low self-esteem [22], and narcissism [23]. Huang et al. [24] reported that negative emotions such as anxiety, stress, and depression significantly predict social media addiction. Furthermore, a predictive role was found for boredom proneness (e.g., [25–27]). For individuals with a high tendency to experience boredom and negative emotions, excessive SNSs use might represent a temporary compensatory strategy to relieve these transient negative states [28, 29].

Among the specific factors involved in high SNSs engagement, the concept of fear of missing out (FoMO) has gained increased attention. FoMO is “a pervasive apprehension that others might be having rewarding experiences from which one is absent” ( [30] p. 1841). In the context of social media users, it reflects preoccupations with the possibility that they have missed satisfying events across their social contacts when not online. FoMO has been positively associated with SNSs use and PSNSU (for a meta-analysis, see [31]). Those who are concerned that they might miss an opportunity for social interaction and/or rewarding experiences happening across their friend networks are more likely to show deficient self-regulation in their use of SNSs because of the need to stay continually connected to what their friends are doing and to alleviate anxious feelings of being socially excluded.

### Theoretical Framework

Cognitive models of addictive behaviors [32] have emphasized the predominant role of desire in activating craving and sustaining addictive behaviors. The Elaborated Intrusion (EI) theory of desire [32–34] suggests for the first time that the conjunction of automatic and voluntary cognitive processes is responsible for the frequency, duration, and intensity of craving. The EI theory of desire postulates that internal (e.g., stress) or external (e.g., viewing a specific image/stimulus related to the desired target) triggers activate the individual thoughts about a desired target or activity (e.g., its positive consequences or sense of deprivation). When the pleasure associated with the desired target/activity or the feeling of deprivation becomes very strong, these associations become conscious, are cognitively elaborated, and provoke the craving experience [35]. The intensification and persistence of craving depend on a cognitive process termed desire thinking (DT) [36–38].

DT is a voluntary and conscious cognitive process oriented to prefigure images, information, and memories about the positive target-related experience [39, 40]. DT comprises two dimensions: Imaginal Prefiguration (IP) deals with the allocation of attentional resources to the information concerning the desired target/activity, and the multi-sensorial elaboration of positive anticipatory imagery or memories related to the desired target/activity (e.g., the individual imagines themself doing the desired activity); Verbal Perseveration (VP) refers to the extended self-talk about the good reasons for engaging and achieving target-related activities (e.g., the individual mentally repeats to themself that they need to practice the desired activity). DT implies a voluntary engagement in elaborating the positive consequences of the desired target/activity, the prolonged self-talk on the good reasons for achieving it, and the mental pianification of actions necessary to reach the desired target/activity [39, 41].

Although in the short-term DT may help to cope with negative emotional states (like the sense of deprivation) by shifting the attention to the positive sensations related to the desired target/activity or by generating a virtual sense of pleasure and relief, in the medium to longer term it drives the experience of craving—an intensely subjective experience that prompts individuals to seek out and achieve a craved target, or practice a dreamed activity, to reach its desired effects [42]—as the target is perseveratively thought about, but not achieved. At this point, the desired target begins to be perceived as the only urgent means to relieve the rising sense of deprivation and craving [40]. Negative consequences of DT include increased craving levels, perception of lack of control, and amplified availability of target-related information [41].

The role of desire thinking in eliciting craving has been extensively studied for substance-related addictive behaviors, especially for Alcohol Use Disorder (e.g., [43]) and smoking behavior (e.g., [44]). More recently, research has enlightened the role of desire thinking in behavioral addictions such as gambling [45], problematic Internet use [46], problematic social media use [47, 48] and problematic Facebook use [49], Internet Gaming Disorder [50], and problematic Internet pornography use [51, 52]. To explain the escalation of DT and craving in addictive behaviors, Spada, Caselli, and Wells [40, 41, 53, 54] have proposed a metacognitive model of desire thinking and craving.

The metacognitive model of DT [41] posits that metacognition plays a central role in understanding dysregulation in DT. Metacognitions about DT refer to the information individuals hold about their own DT and desire-related thoughts. Positive metacognitions regard the usefulness of DT in distracting from negative thoughts and emotions and initiate DT when a target-related thought intrudes into awareness. Negative metacognitions regard the uncontrollability of target-related thoughts and play a role in the propagating of low control once a DT episode has started leading to an escalation of DT and craving.

According to this model, positive metacognitions about desire thinking (PMDT) are associated with imaginal prefiguration (where attentional resources are directed to target-related information) and verbal perseveration (which extends conscious self-talk about reaching the desired activity), marking the activation of desire thinking. Verbal perseveration is associated with negative metacognitions about desire thinking (NMDT) and craving, denoting the pathological escalation of DT. Finally, a direct association between PMDT and NMDT would denote those occasions where target-achieving behavior and perception of low control are not linked with the conscious experience of craving.

The metacognitive model of DT and craving has been validated in clinical (i.e., alcohol use disorder, gambling disorder, problematic internet use, tobacco use disorder) and in a community sample. In the community sample, different from clinical samples, a lack of activation of negative metacognitions about DT as a response to the escalation of craving was found, denoting some qualitative differences [41]. To date, no previous study has empirically tested the metacognitive model of DT and craving in PSNSU.

### The Current Study

**Fig. 1:**
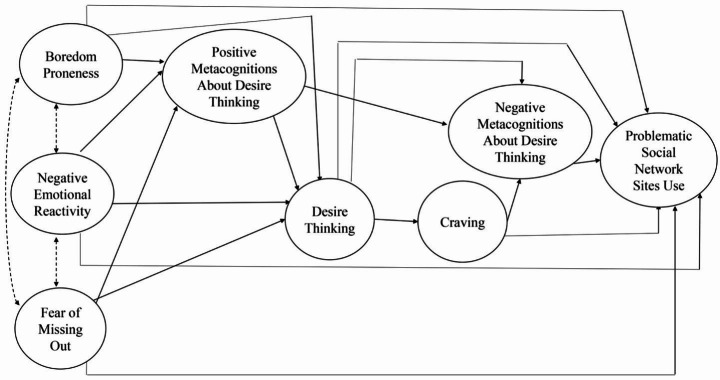
The hypothesized model

The current study aims to fill the identified gap in the literature by evaluating the application of the metacognitive model of desire thinking and craving in PSNSU. In particular, we aimed to test the role of metacognitions about DT, DT, and craving in the relationship between some well-established predisposing psychological factors (i.e., FoMo, boredom proneness, and negative emotional reactivity) and PSNSU. The proposed model is displayed in Fig. 1. Specifically, according to the EI theory of desire and the metacognitive model of desire thinking and craving, we hypothesized that (1) internal triggers (i.e., negative emotional reactivity, FoMo, and boredom proneness) activate thoughts about a desired target or activity (i.e., using SN) through positive metacognitions about DT; (2) DT about SN use is then associated to negative metacognitions about desire thinking and craving related to SNSs use, leading to PSNSU. All the direct paths toward PSNSU would be tested. Age and gender would be considered as control variables as previous studies have shown that women report higher negative emotional reactivity (e.g., [55]) and less boredom proneness than men (e.g., [56]) and women and younger people report higher levels of PSNSU (e.g., [57]).

## Methods

### Participants and Procedure

A sample of 529 participants (*M*_age_ = 32.46 ± 13.34 years; age range = 14–81 years old, %Females = 62.90%) took part in the study. The most reported occupation was worker (43.30%) followed by student (30.40%), working student (20.20%), 3.40% unemployed, and the remaining 2.60% declared to be retired. Concerning educational qualifications, 38.60% of the sample reported having a high school diploma, 27.20% a bachelor’s degree, 20.20% a master’s degree, 7.60% a higher qualification (e.g., Ph.D.), 6% a middle school diploma and the remaining 0.40% an elementary school diploma. Considering participants’ relationship status, 36.70% declared having a cohabiting partner, 32.30% had a non-cohabiting partner, and 31% were single.

Participants were recruited using advertisements on Social Network groups, and they were informed that participation was voluntary and anonymous, and that confidentiality was guaranteed. A web link directed the participants to the study survey. If they consented to participate, it was asked to answer some demographic questions, questions regarding their SNSs use, and a batch of self-report questionnaires. Since it was impossible to submit the form without filling in all the required fields, the results did not present missing data. Data were collected between June 2022 and November 2022, and no remunerative rewards were given. Informed consent was obtained for all participants. The study procedures were carried out in accordance with the Declaration of Helsinki. The Institutional Review Board of the University of Florence approved the study.

### Measures

#### Boredom Proneness

Boredom proneness was assessed using the Italian version [58] of the 8-item Boredom Proneness Scale–Short Form (BPS-SF) [59]. The BPS-SF uses a 7-point Likert scale ranging from 1 (*Highly disagree*) to 7 (*Highly agree*). A sample item is “It takes more stimulation to get me going than most people”. Higher scores indicate higher boredom proneness. In the current sample, Cronbach’s alpha was 0.88, McDonald’s Omega was 0.88, Composite Reliability = 0.91 and Average Variance Extracted (AVE) = 0.55.

#### Negative Emotional Reactivity

Negative emotional reactivity was measured using the 9-item subscale general negative emotional reactivity of the Perth Emotional Reactivity Scale – Short Form (PERS) [60]. Responders are asked to answer on a 5-point Likert Scale ranging from 1 (*Very unlike me*) to 5 (*Very like me)*. A sample item is “Normally when I’m unhappy, I feel it very strongly”. Higher scores indicate higher negative emotional reactivity. In the current sample, Cronbach’s alpha was 0.90, McDonald’s Omega was 0.90, Composite Reliability = 0.91 and AVE = 0.49.

#### Fear of Missing Out

Fear of Missing Out was assessed using the Italian version [61] of the 10-item Fear of Missing Out Scale (FOMOs) [30]. Respondents are asked to indicate how true each statement is for them on a scale from 1 (*Not at all true of me)* to 5 (*Extremely true of me*). An example item includes “I get worried when I find out my friends are having fun without me” and higher scores indicate higher FoMO. In the current sample, Cronbach’s alpha was 0.85, McDonald’s Omega was 0.84, Composite Reliability = 0.90 and AVE = 0.49.

#### Metacognitions About Desire Thinking

Metacognitions about Desire Thinking were measured using the Italian 18-item Metacognitions about Desire Thinking Questionnaire (MDTQ) [62]. Participants were asked to respond on a 4-point Likert scale ranging from 1 (*Highly disagree*) to 4 (*Highly agree*). The questionnaire is composed of three subscales assessing Positive Metacognitive Beliefs about Desire Thinking (PMDT; a sample item is “I need to think about what I desire in order to feel motivated”), Negative Metacognitive Beliefs about Desire Thinking (NMDT; a sample item is “When I begin thinking about a desired activity/object I cannot stop”), and Need to control desire related thoughts (NCDT; a sample item is “Thoughts about certain desires should always be avoided”). Higher scores indicate higher metacognitive thoughts about desire thinking. In the current sample Cronbach’s alphas were 0.78 for PMDT, 0.84 for NMDT, 0.77 for NCDT, McDonald’s Omegas were 0.78 for PMDT, 0.84 For NMDT, 0.78 for NMDT, Composite Reliability was 0.84 for PMDT, 0.88 for NMDT, 0.85 for NCDT, and AVE was 0.40 for PMDT, 0.56 for NMDT, 0.59 for NCDT.

#### Desire Thinking

Desire thinking about Social Networks use was assessed using the Italian 10-item Desire Thinking Questionnaire (DTQ) [40]. Participants are asked to respond on a 4-point Likert scale ranging from 1 (*Almost never)* to 4 (*Almost always*). Higher scores indicate higher levels of desire thinking. A sample item is “I repeat mentally to myself that I need to use Social Networks”; higher scores indicate higher desire thinking. In the current sample, Cronbach’s alpha was 0.90, McDonald’s Omega was 0.90, Composite Reliability = 0.92 and AVE = 0.53.

#### Craving

Craving related to Social Networks Sites use was assessed using the modified version of the 5-item Penn Alcohol Craving Scale (PACS-SNSs) [63] as was previously done in the Italian context by Marino et al. [48]. Participants are asked to respond on a 7-point Likert Scale investigating frequency, intensity, and strength of craving for Social Networks use. Each item addresses experiences within the past week time frame, and a sample item is “During the past week, how often have you thought about Social Networks or how good it makes you feel to check Social Networks?”. Higher scores indicate higher craving. In the current sample, Cronbach’s alpha was 0.85 McDonald’s Omega was 0.85, Composite Reliability = 0.89 and AVE = 0.64.

#### Problematic Social Network Sites Use

Problematic Social Network Sites Use was measured using the Italian version [64] of the 6-items Bergen Social Media Addiction Scale (BSMAS) [65]. Participants give their answers on a 5-point Likert scale ranging from 1 (*Very rarely*) to 5 (*Very often*). Each item addresses experiences within a 12-month period; a sample item is “How often during the last year have you used social networks so much that it has had a negative impact on your job/studies?”. Higher scores indicate higher PSNSU. In the current sample, Cronbach’s alpha was 0.82, McDonald’s Omega was 0.81, Composite Reliability = 0.87 and AVE = 0.53.

### Statistical Analysis

Descriptive statistics and Pearson’s correlations between the study variables were computed. In order to verify the theoretical hypothesized model (Fig. 1), Structural Equation Modeling (SEM) was performed using the lavaan package [66] for the R statistical software (version 4.2.1) with the Maximum Likelihood (ML) estimation method. To limit the number of parameters to be estimated, parcelling was calculated using an empirically equivalent method [67], by assigning items in such a way that the parcels will have equal means, variances, and reliabilities.

To evaluate the model’s goodness of fit, we considered the χ^2^ (and its degrees of freedom and P-value), the Standardized Root Mean Square Residual (SRMR) “close to” 0.09 or lower, the Comparative Fit Index (CFI) “close to” 0.95 or higher, and the Root Mean Square Error of Approximation (RMSEA) less than 0.08 [68]. The indirect effects were tested using the bootstrapping method with 5000 bootstrap samples [69].

## Results

### Descriptive Statistics and Correlational Analyses

Since all the answers were required, the results did not present any missing data.

Regarding participants’ use of Social Networks, the sample reported spending 12.78 ± 9.82 h a week using Social Networks. The Social Networks they declared to use the most were Instagram (52.2%), Facebook (18.9%), and YouTube (16.3%).

Descriptive statistics and Pearson’s correlation among the study variables are presented in Table 1. Significant and positive correlations in the expected direction were found.

**Table 1:** Descriptive statistics and correlations

	M	SD	1.	2.	3.	4.	5.	6.	7.	8.	9.
**1. Boredom Proneness**	23.64	10.18									
**2. Fear of Missing Out**	21.76	7.66	0.51**								
**3. Negative Emotional Reactivity**	27.75	8.54	0.45**	0.45**							
**4. Positive Metacognitions About Desire Thinking**	20.13	4.57	0.25**	0.31**	0.20**						
**5. Negative Metacognitions About Desire Thinking**	14.03	4.42	0.38**	0.39**	0.35**	0.56**					
**6. Need to Control Desire Related Thoughts**	7.46	2.89	0.25**	0.24**	0.23**	0.22**	0.33**				
**7. Desire Thinking**	13.02	4.70	0.39**	0.44**	0.29**	0.33**	0.38**	0.32**			
**8. Craving**	9.06	5.82	0.37**	0.45**	0.22**	0.21**	0.27**	0.18**	0.53**		
**9. Problematic Social Network Sites Use**	11.06	4.67	0.50**	0.52**	0.42**	0.30**	0.38**	0.28**	0.67**	0.64**	

** p < .001

### Structural Equation Modeling

**Fig. 2:**
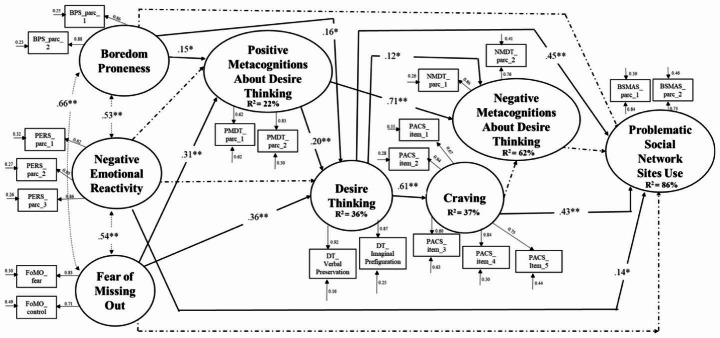
Results of the SEM

The assessed structural model accounted for 86% of the variance of Problematic Social Network Sites Use and showed good fit indices: χ2 = 537.125, df = 185, p < .001; χ2 /df = 2.90; RMSEA [90%CI] = 0.06[0.05-0.07]; CFI = 0.94; SRMR = 0.08. The standardized estimates are depicted in Fig. 2.

In this model, boredom proneness positively predicted PSNSU both directly and indirectly through (i) the serial mediation of positive metacognitions about DT, DT, and craving (β = 0.074, 95% CI [0.023, 0.376]), (ii) the serial mediation of DT and craving (β = 0.043, 95% CI [0.008, 0.225]). Negative emotional reactivity positively predicted PSNSU only directly. Finally, FoMO positively predicted PSNSU only indirectly through (i) the serial mediation of positive metacognitions about DT, DT, and craving (β = 0.016, 95% CI [0.012, 0.077]) (ii) the serial mediation of DT and craving (β = 0.094, 95% CI [0.087, 0.420]). Regarding control variables, being women positively predicted PSNSU, negative emotional reactivity and FoMO, whereas being men positively predicted boredom proneness. Age negatively predicted PSNSU. Given the gender differences that emerged, a multigroup SEM analysis was conducted to evaluate the model invariance across men and women. The fit indices of the model split by gender (configural invariance) seemed acceptable: χ2 = 762.563, df = 346, p < .001; χ2 /df = 2.20; RMSEA [90%CI] = 0.07[0.06-0.08]; CFI = 0.96; SRMR = 0.07. Testing the metric invariance did not seem to deter the model fit significantly: Δ χ2 = 9.789_(12)_ p = .63; Δ CFI = 0.00; Δ RMSEA=-0.007; Δ SRMR = 0.001. Finally, testing the scalar invariance did not seem to worsen the model fit significantly: Δ χ2 = 16.892_(12)_ p = .15; Δ CFI = 0.00; Δ RMSEA=-0.001; Δ SRMR = 0.00. Therefore, the model is independent of gender.

## Discussion

The present study aimed to test the role of metacognitions about desire thinking, DT, and craving in the relationship between some well-established predisposing psychological factors (i.e., FoMo, boredom proneness, and negative emotional reactivity) and PSNSU.

In line with previous studies [47, 49], the role of DT in craving and PSNSU was confirmed. Desire thinking may be a short-term strategy aimed at regulating negative internal states (FoMO and boredom proneness) that fails as, in the medium to longer term, it may lead to an intensification of negative emotions and craving since the desired activity (in this case SNSs use) is perseveringly thought but not achieved [39, 70]. This, in turn, will lead to perceiving SNSs use as the only urgent route to relieve FoMo, boredom, and craving, consequently increasing the probability of engaging in PSNSU as a self-regulation strategy. Furthermore, in the current study, DT is associated with PSNSU indirectly (i.e., through craving mediation) and directly. An explanation may lie in the desired activity (i.e., using SNSs) being easily achievable (for example, via the smartphone), and consequently, craving could be a transient experience.

In a recent study, Brandtner and Brand [70] found that desire thinking mediated the relationship between negative emotional reactivity and craving experience for online activities. Differently, in the current study, we found that the relationship between the tendency to experience negative emotions intensively and PSNSU was not mediated by desire thinking. It is possible to suppose that there might be more important predictors of desire thinking in the context of SNSs use, such as FoMo and boredom proneness. Indeed, when it comes to FoMo, desire thinking fully mediated the relationship between this aversive emotion, craving for SNSs use, and PSNSU. Imagining being on social media to check what friends are doing as a strategy to cope with perceived FoMo seems to be a very relevant path so as to cancel the direct effect of FoMo on craving and problematic use of SNSs.

The current findings provide preliminary evidence for applying the metacognitive model of DT and craving in PSNSU. In particular, a relevant role for positive metacognitions about DT emerged. These beliefs may be involved in initiating DT when SNSs use-related thought intrudes into awareness.

In line with Caselli and Spada’s findings [41], we found a lack of activation of negative metacognitions about DT as a response to the escalation of craving. This result could be explained by the fact that in the current study, we recruited a community sample. This implies a qualitative difference between community and clinical samples that probably lies in how escalating distress is appraised as a signal that may confirm (in clinical samples) the uncontrollability of thoughts and behavior [41]. Having tested the metacognitive model of DT and craving in a community sample could also explain the lack of influence of PMDT on NMDT. As per metacognitive theory [71], positive metacognitions do not necessarily lead to negative metacognitions unless thinking becomes rigid and influenced by the experience of negative affect. In general, negative metacognitions appear predominantly involved in maintaining addictive behaviors, denoting the pathological escalation of desire thinking and craving [72, 73].

The exploration of age and gender differences confirmed previous results concerning the negative association between age and PSNSU (e.g., [57]), the fact that being women positively predicted PSNSU, negative emotional reactivity (e.g., [55]) and FoMO, whereas being men positively predicted boredom proneness (e.g., [56]). However, when we explored potential differences in the structural model across men and women, the model was found to be independent of gender. This result provides preliminary evidence that the same path leading from negative emotional states to PSNSU through the mediation of desire thinking and related metacognition and craving could be involved in both men and women.

### Limitations

This study has several limitations that must be addressed in future research. First, it relies solely on self-report data that are subject to errors in measurement. Second, a cross-sectional design was adopted, which precludes causal inferences. Third, the non-probability sampling method (i.e., convenience sampling) limited the results’ generalizability. Future replications among more representative samples and clinical samples are needed. Finally, the presence of other mental health problems was not examined in the current study. Future research efforts must focus on controlling mental health problems in study samples. Directions for future research should also include ascertaining further the role of desire thinking in predisposition toward, and maintenance of, PSNSU in longitudinal studies (e.g., adopting the Ecological Momentary Assessment procedure). Finally, future research should explore, through experimental designs, the effect of desire thinking induction on the craving experience for SNSs use and the perception of control over own behavior.

## Conclusions

To the best of the authors’ knowledge, the present study is the first to provide preliminary evidence for applying the metacognitive model of desire thinking and craving to PSNSU. In particular, positive metacognitions about DT and DT may be central cognitive processes in craving and PSNSU for individuals who experience boredom proneness and FoMo. Overall, the findings of the present study suggest that, as for other addictive behaviors, adopting a metacognitive framework might prompt the understanding of the mechanisms that underpin addiction-like behaviors in relation to SNS. In particular, if future studies with more accurate designs will confirm the current results, several implications for the assessment and treatment of PSNSU may be drawn. In terms of assessment, it may be helpful to gather information not only in relation to negative affect and the experience of craving for SNSs use but also to desire thinking and related metacognitions. With respect to interventions, interventions aimed at attenuating the propensity to engage in desire thinking and restructuring metacognitions about the usefulness of DT to regulate emotional states might be helpful in reducing the risk of PSNSU. This could be achieved, for example, by providing information on the role of desire thinking in both the activation of craving and the excessive and uncontrolled use of the SNSs. Furthermore, applying Metacognitive Therapy techniques, like attention training and detached mindfulness, could be used to increase the level of flexible control over attention and thinking style as well as for questioning beliefs about the benefits of desire thinking aimed at modifying dysfunctional metacognitions that increase the intensity of DT and craving in the context of PSNSU.

## Data Availability

The data that support the findings of this study are available from the corresponding author, [G.F.], upon reasonable request.
